# Enhancing the Accuracy and Robustness of a Compressive Sensing Based Device-Free Localization by Exploiting Channel Diversity

**DOI:** 10.3390/s19081828

**Published:** 2019-04-17

**Authors:** Dongping Yu, Yan Guo, Ning Li, Xiaoqin Yang

**Affiliations:** College of Communications Engineering, Army Engineering University of PLA, Nanjing 210007, China; yudongpingxm@126.com (D.Y.); lining_friend@sina.com (N.L.); 15261856573@139.com (X.Y.)

**Keywords:** changing environment, channel diversity, variational Bayesian inference, joint sparse recovery, device-free localization

## Abstract

As an emerging and promising technique, device-free localization (DFL) estimates target positions by analyzing their shadowing effects. Most existing compressive sensing (CS)-based DFL methods use the changes of received signal strength (RSS) to approximate the shadowing effects. However, in changing environments, RSS readings are vulnerable to environmental dynamics. The deviation between runtime RSS variations and the data in a fixed dictionary can significantly deteriorate the performance of DFL. In this paper, we introduce ComDec, a novel CS-based DFL method using channel state information (CSI) to enhance localization accuracy and robustness. To exploit the channel diversity of CSI measurements, the DFL problem is formulated as a joint sparse recovery problem that recovers multiple sparse vectors with common support. To solve this problem, we develop a joint sparse recovery algorithm under the variational Bayesian inference framework. In this algorithm, dictionaries are parameterized based on the saddle surface model. To adapt to the environmental changes and different channel characteristics, dictionary parameters are modelled as tunable parameters. Simulation results verified the superior performance of ComDec as compared with other state-of-the-art CS-based DFL methods.

## 1. Introduction

As a main piece of the context information, location information is essential for the location-based services (LBS) (all the used abbreviations are explained at the end of the article) in many context-aware applications. Nowadays, localization techniques have been an active field in pervasive and ubiquitous computing. Generally, the target localization methods can be classified as: device-free [1,2,3] and device-based [4,5,6]. Device-based methods need objects to attach assistant wireless devices for signal transmitting or receiving. However, in many applications, such as emergency rescue, intruder detection, and smart homes, it is difficult to attach objects with any transceivers. In this case, the device-based method will be infeasible.

Device-free localization (DFL) methods do not need to attach objects with any assistant devices. This approach has become a crucial component in many context-aware applications. As the transceiver-free objects cannot be directly perceived, DFL methods estimate their positions by analyzing their influences on surrounding radio environments. Among current DFL methods, some rely on specialized hardware, such as radar-based methods [7], camera-based methods [8], and infrared-based methods [9]. These methods need an extensive deployment of dedicated devices in the monitoring area and may involve privacy issues. In recent years, DFL methods based on existing infrastructures (e.g., WSNs/WiFi) have attracted a lot of research interests. They do not require dedicated hardware and only make use of the measurement information available from the already deployed wireless devices.

As a common link measurement, the received signal strength (RSS) is nearly ubiquitously available from the standard radio transceivers [10,11]. The RSS measurement information has been widely used in DFL. When targets located at different positions, the RSS readings will be different. To exploit the location dependence of RSS, RSS readings on multiple wireless links are recorded before and after targets entering into the monitoring area. To establish the relationship between RSS variations and target locations, the fingerprinting technique has been introduced [12]. Typically, the fingerprinting-based DFL consists of an online and offline phase. In the offline training phase, by gathering the RSS variations caused by a target at every possible location, a radio map can be built. In the online locating phase, target locations are estimated by matching current RSS variations with the fingerprints in radio map. However, as a major drawback of the fingerprinting-based DFL methods, gathering fingerprints to build a radio map is labor-intensive and time-consuming.

As an alternative to fingerprinting-based methods, model-based methods have been widely used in DFL. They map RSS variations to target positions by theoretical or empirical shadowing models. Based on these models, the dictionary (a.k.a. radio map) can be built without site survey. Unfortunately, in changing environments, RSS is extremely sensitive to environmental dynamics, such as temperature, humidity, electromagnetic characteristics, and pedestrians around [13]. In this case, shadowing effects cannot be well approximated by RSS variations. The spatial and temporal environmental dynamics may result in mismatches between runtime RSS measurements and the data in a fixed dictionary. In [14], a dictionary refinement algorithm is introduced, which alleviates the dictionary mismatches by iteratively refining the model-based dictionary. However, the real-time optimization of dictionary parameters leads to a high computational complexity. To avoid this, in ComDec, the dictionary parameters are modelled as tunable parameters to adapt to the changes of environment, and we do not need to explicitly estimate their values.

Recent years, in target localization, the channel state information (CSI) [15] has been exploited, which is a measurement information from PHY layer. As a fine-grained value, CSI depicts the channel quality on multiple orthogonal subcarriers. In wireless environments, due to the frequency-independent attenuation and frequency-selective fading, each channel will exhibit a unique amplitude and phase. Hence, the dictionaries corresponding to different channels are different. Over the past few years, in many device-based localization methods, CSI measurements has been leveraged [16]. Recently, more efforts have been paid in device-free technique. In [17], the fine-grained subcarrier information is exploited based on the multitask Bayesian compressive sensing (MBCS). However, the DFL method proposed in [17] do not provide a solution to the dictionary mismatch problem caused by environmental dynamics.

Compared with RSS measurements, CSI measurements are more robust and suitable for being utilized in DFL. In this paper, a novel ComDec method is proposed. It can enhance the accuracy and robustness of the CS-based DFL in changing environments. In ComDec, to enrich the measurement information, CSI measurements are collected from multiple frequency bands. Moreover, to reduce wireless sensor nodes, the compressive sensing (CS) theory [18] is applied in ComDec by taking advantage of the spatial sparsity of target localization. The CS-based DFL problem in multi-channel scenario is formulated as a joint sparse recovery problem. Furthermore, to bypass the dictionary training and retraining works, the dictionaries are built based on the saddle surface model [19]. We treat dictionary parameters as tunable parameters to adapt the changes of environment and different channel characteristics. Afterwards, to recover the sparse vectors of multiple channels, we develop a joint sparse recovery algorithm under the variational Bayesian inference framework [20]. The main contributions of this work are as follows:

- To enhance the localization accuracy and robustness of CS-based DFL, a novel ComDec method is proposed, which leverages the channel diversity of CSI measurements. In ComDec, the CS-based DFL problem is extended to multi-channel scenario. It is formulated as a joint sparse recovery problem that recovers multiple jointly sparse vectors over two known dictionaries.
- To simultaneously recover the jointly sparse vectors, we develop a novel joint sparse recovery algorithm. The joint sparsity of the sparse vectors is induced by a novel two-layer hierarchical prior model. Then, the common support set of the sparse vectors is estimated by inferring the posteriors of the hidden variables that defined in the proposed prior model.
- To mitigate the influence of environmental dynamics in changing environments, the dictionary parameters with respect to multiple channels are modelled as tunable parameters to adapt the environmental changes and different channel characteristics. In this way, the dictionary mismatch problem can be solved without the need of explicitly estimating the dictionary parameters.
- To reduce the computational complexity, we introduce four methods in the proposed joint sparse recovery algorithm. Among them, the grid pruning method can improve the convergence speed of the proposed joint sparse recovery algorithm.

The remainder of this paper is organized as follows. Section 2 presents an overview of the related works on multi-target DFL. Section 3 gives the signal model and formulates the CS-based DFL problem as a joint sparse recovery problem. Section 4 proposes a novel joint sparse recovery algorithm to recover the jointly sparse vectors. Section 5 validates the proposed ComDec method with extensive numerical simulations. Finally, Section 6 concludes this paper.

## 2. Related Work

Beginning with the initial papers of Youssef et al. [1] and Zhang et al. [2], numerous research works on DFL have been carried out [17,21]. Typically, there are four types of DFL methods: (1) geometry-based methods, (2) fingerprinting-based methods, (3) radio tomographic imaging (RTI)-based methods, and (4) CS-based methods. The geometry-based methods estimate target locations based on the geometry information of wireless links. They have a restriction on target spacing and require to know the deployment information of wireless nodes. Fingerprinting-based methods estimate target locations by matching runtime measurements with fingerprints in a radio map. They can achieve enhanced accuracy, but a site survey is needed for radio map building and retraining. RTI-based methods do not require offline training effort. They treat target locations as the attenuation images of distorted links and can achieve an improved performance. However, they need a dense deployment of wireless links. It may lead to high hardware cost and great energy consumption. CS-based methods formulate the DFL problem as a sparse signal reconstruction problem. Compared with the aforementioned DFL methods, the CS-based DFL methods can ensure a high accuracy with fewer measurements. LCS [22] is a CS-based DFL method, in which the model-based dictionary satisfies the restricted isometry property (RIP) with high probability. E-HIPA [23] is a representative CS-based DFL method. It adopts an adaptive orthogonal matching pursuit (OMP) algorithm to estimate the target number and location vector. DR-DFL [14] introduced a real-time dictionary refinement algorithm for CS-based DFL. It can mitigate the influence of environmental dynamics in changing environments. The dictionary refinement is realized by optimizing the environment-related dictionary parameters. However, explicitly estimating the dictionary parameters may lead to a high computational complexity.

To leverage CSI for DFL, Pilot [24] regards the correlations of CSI as fingerprints and uses the maximum a posteriori probability (MAP) estimator to estimate target location. MonoPHY [25] is a fingerprinting-based DFL method that leverages CSI to locate a target with only one stream. Gao et al. [26] transformed CSI measurements into a radio image and adopted the machine learning method to estimate the position of a person. The aforementioned DFL method leverage CSI by fingerprinting technique. They seek appropriate location-dependent CSI features for DFL and trying to build a robust and precise relationship between CSI measurements and target position. However, to make full use of the channel diversity of CSI measurements is still a challenging issue in CS-based DFL.

## 3. Preliminaries and Problem Formulation

### 3.1. Overview of Multi-Target Device-Free Localization

**Figure 1:**
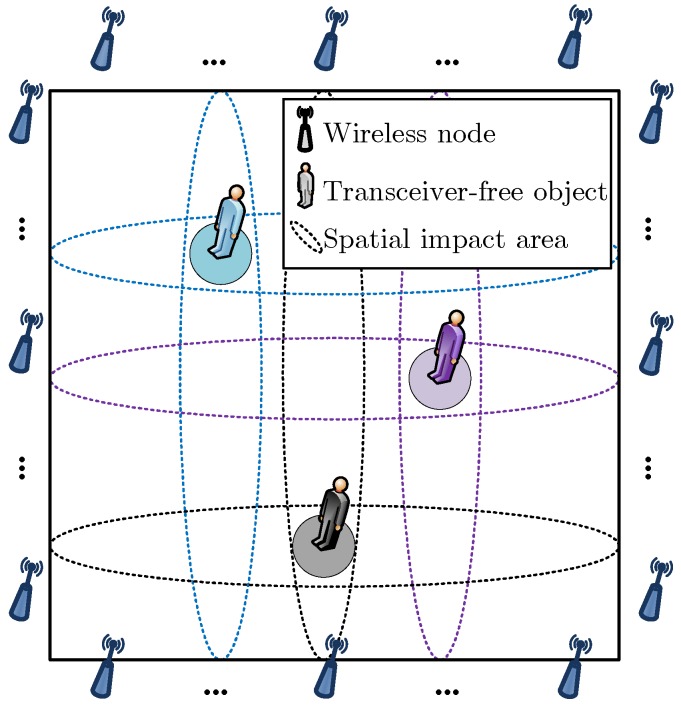
An example of the CS-based DFL.

The proposed ComDec method is a CS-based DFL method that aims to estimate the number and locations of multiple transceiver-free objects in changing environments. Figure 1 shows an example of the CS-based DFL. As can be seen, multiple transceiver-free objects are randomly distributed in an l×l two-dimensional (2D) monitoring area A. Owing to the inherent spatial sparsity of the targets in A, the position information of multiple targets can be considered as a sparse signal. To achieve this, we discretize A into *N* equal-sized grids. When a target is located in grid *n*, we regard the grid center as its position. In this case, the target number and position information can be encoded in a sparse location vector, i.e.,

(1) $$\theta ={\left[\theta_{1},\theta_{2},...,\theta_{n},...,\theta_{N}\right]}^{T},$$

where $\theta \in R^{N\times 1}$ is a *K*-sparse vector. $\theta_{n}\in \left\{0,1\right\}$ is its *n*-th component, which indicates whether a target is located in grid *n*. If there is a target, $\theta_{n}=1$; otherwise, $\theta_{n}=0$. Thus, the location information of *K* targets can be denoted as $L=\left\{\left(x_{n},y_{n}\right)\left|\theta_{n}=1,n\in \left\{1,...,N\right\}\right.\right\}$, where $\left(x_{n},y_{n}\right)$ is the coordinate of grid *n*. Moreover, the target number can be denoted as $K={∥\theta ∥}_{0}$. When the target number *K* is far less than the grid number *N* (K≪N), the sparsity of θ can be ensured. As a matter of fact, the grid number *N* is usually a big number, while there is only a small number of targets in the monitoring area.

As seen from Figure 1, A is surrounded by several wireless nodes. Between these nodes, *M* bidirectional wireless links are established to cover A. They travel through A and sense the target-induced shadowing effects in the electromagnetic field. When there exists a target, some of the wireless links will be shadowed, and the signal power or other features of radio signals will be affected due to the scattering, reflection, refraction, and absorption of the signals. Fortunately, the changes of the signal features are closely related to target positions. Therefore, by analyzing the target-induced shadowing effects, the location information of multiple targets can be inferred.

### 3.2. CSI Collection and Feature Extraction

RSS is a coarse-grained measurement information from MAC layer. It represents the overall signal power across all subchannels. Due to the multipath fading, RSS is unreliable and varies with time even in a static environment. This may result in limited localization accuracy, especially in changing environments. To enhance the localization performance of CS-based DFL, ComDec adopts CSI to characterize target-induced perturbations. CSI is a fine-grained measurement information from PHY layer. It can provide the phase and strength information of the signals on different subchannels. For link *m*, the CSI on each subchannel is a complex value which is defined as

(2) $$H_{m}^{f}=\left|H_{m}^{f}\right|e^{j\sin\left\{∠H_{m}\right\}},\;\;f=1,2,...,F,$$

where $H_{m}^{f}$ denotes the CSI measurement corresponding to the *f*-th channel of link *m*. $|H_{m}^{f}|$ and $∠H_{m}$ denote the amplitude and phase response, respectively. *F* is the number of channels. The amplitude response $|H_{m}^{f}|$ is the change of amplitude of link *m* on channel *f*. By converting it from linear space to logarithmic space, the corresponding power fading can be written as ${\tilde{H}}_{m}^{f}=20\log\left(|H_{m}^{f}|/10^{3}\right)$ (dBm) [27]. We collect the CSI measurements from *F* channels, and a set of power fading information with channel diversity can be obtained. They can provide redundancy information to alleviate the location ambiguity that is incurred by environmental dynamics.

### 3.3. Problem Formulation

To characterize shadowing effects, we measure the the change of power fading on each link. For each channel, the change of power fading on link *m* is

(3) $$\begin{array}{c} \Delta {\tilde{H}}_{m}^{f}={\tilde{H}}_{m}^{f}-{\tilde{H}}_{m}^{f}\left(0\right)\approx S_{m}^{f}+\epsilon_{m}^{f}, \end{array}$$

where ${\tilde{H}}_{m}^{f}$ denotes the current power fading, ${\tilde{H}}_{m}^{f}\left(0\right)$ is the reference power fading recorded when A is vacant, $S_{m}^{f}$ represents the target-induced shadowing loss, and $\epsilon_{m}^{f}$ denotes the measurement noise.

The changes of power fading are measured on *M* links at runtime. For each link, we can obtain *F* measurements from *F* channels. Thus, the measurement information can be represented as

(4) $$Y=\left[y^{1},y^{2},...,y^{f},...,y^{F}\right]={\left[\begin{array}{cccc} y_{1}^{1} & y_{1}^{2} & \cdots & y_{1}^{F}\\ y_{2}^{1} & y_{2}^{2} & \cdots & y_{2}^{F}\\ \vdots & \vdots & \ddots & \vdots \\ y_{M}^{1} & y_{M}^{2} & \cdots & y_{M}^{F} \end{array}\right]}_{M\times F,}$$

where $Y\in R^{M\times F}$ is the measurement matrix. $y^{f}\in R^{M\times 1}$ is the *f*-th column of Y. Its *m*-th component $y_{m}^{f}=\Delta {\tilde{H}}_{m}^{f}$.

If any two targets are located sparsely [23] in the monitoring area, $S_{m}^{f}$ can be represented as the summation of attenuations that occur in each cell [28]. Therefore, $\Delta {\tilde{H}}_{m}^{f}$ can be expressed as

(5) $$\Delta {\tilde{H}}_{m}^{f}=\sum_{n=1}^{N}\theta_{n}\cdot {\tilde{h}}_{m,n}^{f}+\epsilon_{m}^{f},$$

where ${\tilde{h}}_{m,n}^{f}$ is the shadowing loss that caused by a target located in grid *n*. For *M* links, the measurement vector $y^{f}$ can be written as

(6) $$y^{f}=\Phi^{f}\theta +\epsilon^{f},$$

where $\Phi^{f}\in R^{M\times N}$ is a dictionary. Its (m,n)-th element is ${\tilde{h}}_{m,n}^{f}$. $\epsilon^{f}\in R^{M\times 1}$ represents the noise vector of channel *f*, and its *m*-th component is $\epsilon_{m}^{f}$. In ComDec, the DFL problem can be viewed as a problem of reconstructing the location vector θ from the measurements Y. Theoretically, this problem can be solved by existing joint sparse recovery methods. Nonetheless, as a common shortcoming, they require the true dictionaries ${\left\{\Phi^{f}\right\}}_{f=1}^{F}$ to be known in advance. However, in changing environments, it is impossible for us to accurately estimate these dictionaries.

Due to the difference in channel characteristics, the dictionaries with respect to different channels are different. Furthermore, in changing environments, the dictionary for an individual channel may differ when observed at different times. In this case, the fingerprinting method will be infeasible because it may cumulate the effects of dictionary mismatches on multiple channels and deteriorate the localization performance significantly. As an alternative, we adopt the saddle surface model to characterize the shadowing effect and establish multiple model-based dictionaries for multiple channels. To adapt to the changes of environment and different channel characteristics, the environmental parameters in these dictionaries are considered as adjustable. For simplicity, we denote $\phi_{m,n}^{f}={\tilde{h}}_{m,n}^{f}$, and thus $\Phi^{f}$ can be expressed as

(7) $$\Phi^{f}={\left[\phi_{1}^{f},\phi_{2}^{f},...,\phi_{m}^{f},...,\phi_{M}^{f}\right]}^{T}={\left[\begin{array}{cccc} \phi_{1,1}^{f} & \phi_{1,2}^{f} & \cdots & \phi_{1,N}^{f}\\ \phi_{2,1}^{f} & \phi_{2,2}^{f} & \cdots & \phi_{2,N}^{f}\\ \vdots & \vdots & \ddots & \vdots \\ \phi_{M,1}^{f} & \phi_{M,2}^{f} & \cdots & \phi_{M,N}^{f} \end{array}\right]}_{M\times N.}$$

**Figure 2:**
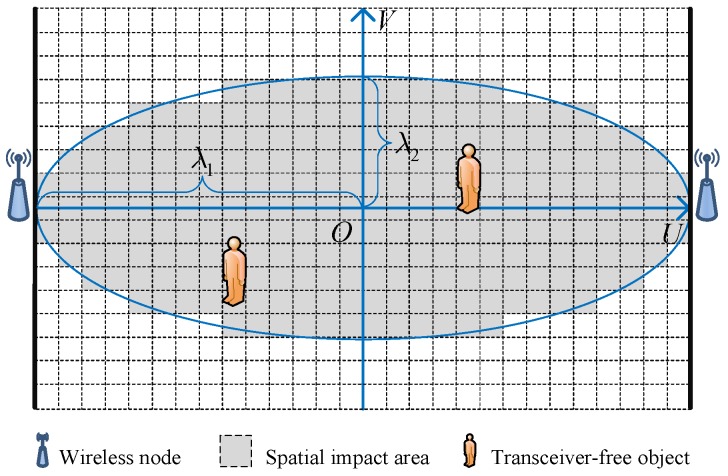
Spatial impact area of a wireless link.

Figure 2 depicts the spatial impact area of a wireless link. As can be seen, the spatial impact area is an ellipse area, and we set up an *U*-*V* coordinate system in the area. Based on the saddle surface model, $\phi_{m,n}^{f}$ can be expressed as

(8) $$\begin{array}{c} \phi_{m,n}^{f}=\gamma_{m}^{f}\cdot \left(\frac{1-\rho_{m}^{f}}{\lambda_{1}^{2}}U_{m,n}^{2}+\rho_{m}^{f}\cdot \left(1-\frac{V_{m,n}^{2}}{\lambda_{2}^{2}}\right)\right),\\ \;\;\;\;\;\;\;\;\;\;s.t.\;\;\;\;\frac{U_{m,n}^{2}}{\lambda_{1}^{2}}+\frac{V_{m,n}^{2}}{\lambda_{2}^{2}}⩽1, \end{array}$$

where $\left(U_{m,n},V_{m,n}\right)$ denotes the coordinate of grid *n*. $\lambda_{1}$ and $\lambda_{2}$ denote the semi-major and semi-minor axes of the spatial impact area, respectively. In the saddle surface model, $\gamma_{m}^{f}$ represents the maximum shadowing loss, and $\rho_{m}^{f}\in \left(0,1\right]$ denotes the shadowing ratio, which represents the normalized shadowing loss at the midpoint of LOS path.

Due to A being an isotropic free space and all links sharing a common link length, we have $\gamma_{m}^{f}=\gamma^{f}$ and $\rho_{m}^{f}=\rho^{f}$. This means that the values of $\gamma^{f}$ and $\rho^{f}$ are only determined by the environmental characteristics. In this case, the shadowing loss $\phi_{m,n}^{f}$ can be rewritten as

(9) $$\phi_{m,n}^{f}=\frac{U_{m,n}^{2}}{\lambda_{1}^{2}}\gamma^{f}+\gamma^{f}\rho^{f}\left(1-\frac{U_{m,n}^{2}}{\lambda_{1}^{2}}-\frac{V_{m,n}^{2}}{\lambda_{2}^{2}}\right).$$

Let $\omega^{f}=\gamma^{f}\cdot \theta$ and $\upsilon^{f}=\gamma^{f}\rho^{f}\cdot \theta$ be the unknown sparse vectors. Based on (6) and (9), $y^{f}$ can be rewritten as

(10) $$y^{f}=\Psi \omega^{f}+\Psi^{\prime }\upsilon^{f}+\epsilon^{f},$$

where $\Psi \in R^{M\times N}$ and $\Psi^{\prime }\in R^{M\times N}$ are known dictionaries. Their (m,n)-th elements are computed as ψm,n=Um,n2Um,n2λ12λ12 and ψm,n′=1−Um,n2Um,n2λ12λ12−Vm,n2Vm,n2λ22λ22, respectively. We can determine their values before the localization stage according to the deployment of wireless links. It is noteworthy that the location vector θ and sparse vectors $S={\left\{\omega^{f},\upsilon^{f}\right\}}_{f=1}^{F}$ share a common sparse support set T⊆{1,2,...,N}. In other words, their nonzero entries are concentrated at some common locations.

As pointed out earlier, the values of ${\left\{\gamma^{f},\rho^{f}\right\}}_{f=1}^{F}$ are closely related to the environmental characteristics, which are different for different channels and times. Let $\gamma =\left[\gamma^{1},\gamma^{2},...,\gamma^{F}\right]$ and $\rho =\left[\rho^{1},\rho^{2},...,\rho^{F}\right]$. We define two matrices $\Theta ,\Theta^{\prime }\in R^{N\times F}$, which are constructed as follows:

(11) $$\Theta =\theta \gamma =[\omega^{1},\omega^{2},...,\omega^{F}],$$

(12) $$\Theta^{\prime }=\theta \left(\gamma ⊙\rho \right)=\left[\upsilon^{1},\upsilon^{2},...,\upsilon^{F}\right].$$

Both Θ and $\Theta^{\prime }$ are *K* jointly sparse matrices. This means there are at most *K* rows in them that have nonzero elements. The column vectors of Θ and $\Theta^{\prime }$ share the common support T, which is the index set of the grids where a target exists. The cardinality of T is $K:=\left|\mathrm{supp}\Theta \right|=\left|\mathrm{supp}\Theta^{\prime }\right|$, which also denotes the target number. Based on (10), (11) and (12), the measurement matrix Y can be expressed as

(13) $$Y=\Psi \Theta +\Psi^{\prime }\Theta^{\prime }+E,$$

where $E\in R^{M\times F}$ is the matrix of measurement noises, whose *f*-th column is $\epsilon^{f}$. Θ and $\Theta^{\prime }$ are the unknown matrices. By (13), the CS-based DFL problem is recast as a problem that needs to recover Θ and $\Theta^{\prime }$ simultaneously over two known dictionaries. It should be pointed out that the problem is different from the conventional joint sparse recovery problem, which only needs to reconstruct a single sparse matrix over a single sparsifying dictionary. However, in this problem, there are two different sparse matrices to be estimated. Therefore, existing joint sparse recovery algorithms cannot be directly applied in ComDec. In this context, the key issue of ComDec is to design a joint sparse recovery algorithm to estimate Θ and $\Theta^{\prime }$ simultaneously. It should be noted that, the channel diversity of CSI measurements can be exploited to enhance the accuracy and robustness of the joint sparse recovery.

**Figure 3:**
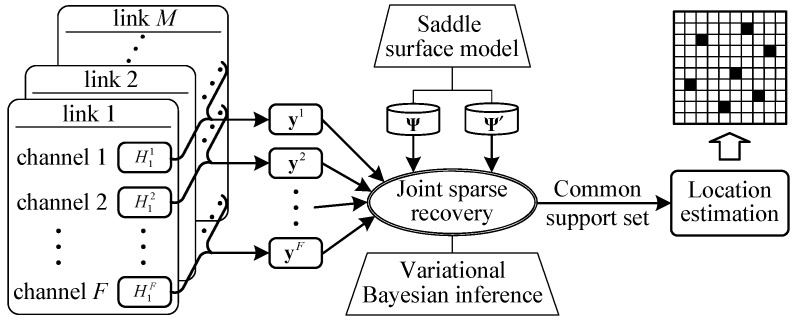
Architecture of the ComDec method.

Figure 3 shows an architectural overview of ComDec. The proposed ComDec consists of four main modules: dictionary construction, measurement information collection, joint sparse recovery, and location estimation. The process of target counting and localization is illustrated as follows: First, in the dictionary construction module, we establish two constant dictionaries Ψ and $\Psi^{\prime }$ according to the saddle surface model. Then, at runtime, CSI measurements are collected from *M* links. Each stream contains the CSI readings of *F* channels. Afterwards, in the joint sparse recovery module, the posteriors of all hidden variables are inferred by a joint sparse recovery algorithm. Finally, with the knowledge of the posteriors, in the location estimation module, the common support set T can be estimated, and thus the estimated Cartesian coordinates of multiple targets can be obtained.

## 4. Target Localization via Variational Bayesian Inference

In this section, we develop a novel joint sparse recovery algorithm. First, to induce the joint sparsity of the jointly sparse vectors S, a two-layer hierarchical prior model is introduced. Then, by using the variational Bayesian inference technique, we infer the posteriors of the hidden variables that defined in the proposed hierarchical prior model. Finally, based on the posteriors of S, target counting and localization are implemented.

### 4.1. Hierarchical Prior Model

**Figure 4:**
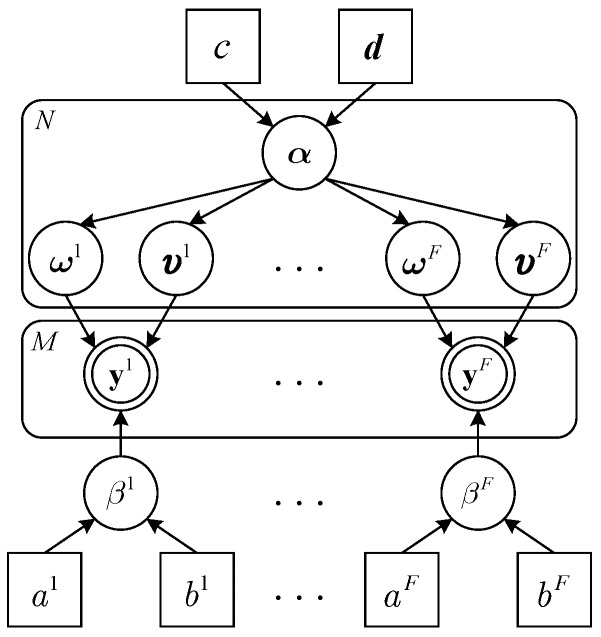
Graphical model for the joint sparse recovery.

The joint sparsity of S is induced by a non-separable sparsity inducing prior model [29]. The graphical model for the joint sparse recovery is shown in Figure 4, which describes the dependencies between random variables. In the first layer, we regard ${\left\{\omega^{f},\upsilon^{f}\right\}}_{f=1}^{F}$ as stochastic variables and define $\omega ={\left\{\omega^{f}\right\}}_{f=1}^{F}$ and $\upsilon ={\left\{\upsilon^{f}\right\}}_{f=1}^{F}$. Moreover, the Gaussian-inverse-Gamma prior is imposed on each sparse vector to encourage its sparsity. $\omega^{f}$ is treated as a Gaussian random variable, whose prior distribution is can be given as

(14) p(ωfα)=∏n=1NNωnf0,αn−1=2π−N−N22Λ1/2exp12(ωf)TΛωf,

where $N\left(\cdot \left|0,\alpha_{n}^{-1}\right.\right)$ denotes the Gaussian distribution with zero mean and variance of $\alpha_{n}^{-1}$. $\alpha_{n}$ is the inverse variance (precision) of ${\left\{\omega_{n}^{f}\right\}}_{f=1}^{F}$. We define $\alpha ={\left[\alpha_{1},\alpha_{2},...,\alpha_{N}\right]}^{T}$ and $\Lambda =\mathrm{diag}\left(\alpha \right)$. As $\upsilon^{f}=\rho^{f}\omega^{f}$, where $\rho^{f}$ is regarded as an unknown deterministic parameter, a multivariate Gaussian prior is also imposed on $\upsilon^{f}$. The variance of $\upsilon_{n}^{f}$ can be given as ${\left(\rho^{f}\right)}^{2}\alpha_{n}^{-1}$. We set $\rho^{f}=1$ to accommodate the worst case of the variance. In this situation, the prior of $\upsilon^{f}$ can be expressed as

(15) $$p\left(\upsilon^{f}\left|\alpha \right.\right)=\prod_{n=1}^{N}N\left(\upsilon_{n}^{f}\left|0,\alpha_{n}^{-1}\right.\right).$$

The variances of $\upsilon_{n}^{f}$ and $\omega_{n}^{f}$ are identical. Obviously, a large value of $\alpha_{n}$ will simultaneously drive $\upsilon_{n}^{f}$ and $\omega_{n}^{f}$ to zero, which is in correspondence with the joint sparsity between $\upsilon^{f}$ and $\omega^{f}$. Intuitively, if most of the components of α have large values, the jointly sparse solutions will be obtained. Hence, we model the hyperparameters ${\left\{\alpha_{n}\right\}}_{n=1}^{N}$ as hidden variables to allow the flexibility to learn and adapt to the true situation.

To allow conjugate-exponential analysis [20], an independent identically distributed (i.i.d.) Gamma distribution is imposed on α, which can be expressed as

(16) $$p\left(\alpha ;c,d\right)=\prod_{n=1}^{N}\mathrm{Gamma}\left(\alpha_{n}\left|c,d_{n}\right.\right)=\prod_{n=1}^{N}\frac{1}{\Gamma \left(c\right)}d_{n}^{c}\alpha_{n}^{c-1}\exp\left(-d_{n}\alpha_{n}\right),$$

where $\mathrm{Gamma}\left(\cdot \left|c,d_{n}\right.\right)$ denotes the Gamma distribution with parameters *c* and $d_{n}$, $\Gamma \left(c\right)=\int_{0}^{\infty }x^{c-1}e^{-x}dx$ is the Gamma function, $d={\left[d_{1},d_{2},...,d_{N}\right]}^{T}$. We set the hyperparameters *c* and $d_{n}$ to very small values (e.g., $10^{-6}$) to provide a non-informative hyperprior over $\alpha_{n}$. The Gamma distribution is generally chosen as the prior for the inverse variance of a Gaussian distribution, because it is the conjugate prior of the Gaussian distribution. In this case, the associated Bayesian inference can be performed in closed form [4].

When a hierarchical Gaussian prior model imposed on $\omega^{f}$, the true prior distribution of $\omega^{f}$ can be computed by marginalizing the parameter α, i.e.,

(17) $$\begin{array}{cc} p\left(\omega^{f};c,d\right) & =\int p(\omega^{f}|\alpha )p\left(\alpha ;c,d\right)d\alpha \\ & =\int \prod_{n=1}^{N}N\left(\omega_{n}^{f}|0,\alpha_{n}^{-1}\right)\mathrm{Gamma}\left(\alpha_{n}|c,d\right)d\alpha_{n}\\ & =\prod_{n=1}^{N}\mathrm{St}(\omega_{n}^{f}|\lambda ,v). \end{array}$$

In this case, the true distribution of $\omega^{f}$ is a Student-t pdf,

(18) St(x|κ,λ,v)=Γ((v+1)(v+1)22)Γ(vv22)λπv11221+λ(x−κ)2v−(v+1)−(v+1)22,

with mean κ=0, parameter λ=ccdd and degrees of freedom v=2c. According to the property of the Student-t distribution, when *v* is small, this distribution will exhibit very heavy tails. Thus, it favours sparse solutions, which include only few of the basis functions and prunes the remaining basis functions by setting the corresponding weights to very small values. In this case, a sparse vector $\omega^{f}$ can be induced with the hierarchical Gaussian prior model. As the case of $\omega^{f}$, we can also induce the sparsity of $\upsilon^{f}$ by the proposed hierarchical Gaussian prior model.

As illustrated earlier, $\epsilon^{f}$ follows an i.i.d. Gaussian distribution. The prior distribution of $\epsilon^{f}$ is defined as

(19) $$p\left(\epsilon^{f}|\beta^{f}\right)=\prod_{n=1}^{N}N\left(\epsilon_{m}^{f}\left|0,{\left(\beta^{f}\right)}^{-1}\right.\right)={\left(\frac{\beta^{f}}{2\pi }\right)}^{\frac{M}{2}}\exp\left(-\frac{\beta^{f}}{2}{∥\epsilon^{f}∥}_{2}^{2}\right),$$

where $\beta^{f}$ is the inverse variance of $\epsilon^{f}$. We treat $\beta^{f}$ as a hidden variable and define $\beta ={\left\{\beta^{f}\right\}}_{f=1}^{F}$. As $\epsilon^{f}$ follows a Gaussian distribution, a Gamma prior is also imposed on each $\beta^{f}$, i.e.,

(20) $$p\left(\beta^{f};a^{f},b^{f}\right)=\mathrm{Gamma}\left(\beta^{f}|a^{f},b^{f}\right)=\frac{{\left(b^{f}\right)}^{a^{f}}{\left(\beta^{f}\right)}^{a^{\left(f\right)}-1}}{\Gamma \left(a^{f}\right)}\exp\left(-b^{f}\beta^{f}\right),$$

where $a^{f}$ and $b^{f}$ are deterministic parameters of the Gamma distribution. We denote $a={\left\{a^{f}\right\}}_{f=1}^{F}$ and $b={\left\{b^{f}\right\}}_{f=1}^{F}$. To assume uninformative priors for β, the hyperparameters a and b are also set to very small values (e.g., $10^{-6}$).

In the proposed graphical model, the observed variables are $y={\left\{y^{f}\right\}}_{f=1}^{F}$, and the hidden variables are $z≜\left\{\alpha ,\omega ,\upsilon ,\beta \right\}$. To estimate the jointly sparse vectors, we need to infer the posterior distributions of z based on the predefined prior evidence and the measurement data. In addition, the deterministic parameters of the prior model are $\Omega ≜\left\{c,d,a,b\right\}$, which are fixed at small values to allow uninformative hyperpriors for α and β.

### 4.2. Variational Bayesian Inference

In the joint sparse recovery module, the key task is to infer the posteriors of z. Afterwards, based on these posteriors, the target number and locations can be estimated. For this objective, the variational Bayesian inference technique is adopted, which is applied due to it can deal with complicated Bayesian models [20]. Based on (10) and (19), the likelihood function of channel *f* can be written as

(21) $$p\left(y^{f}|\omega^{f},\upsilon^{f},\beta^{f}\right)={\left(\frac{\beta^{f}}{2\pi }\right)}^{\frac{M}{2}}\exp\left(-\frac{\beta^{f}}{2}{∥y^{f}-\Psi \omega^{f}-\Psi^{\prime }\upsilon^{f}∥}_{2}^{2}\right).$$

According to the chain rule of probability, the joint probability density function (PDF) of y and z can be written as

(22) $$\begin{array}{cc} p\left(y,z\right) & =p\left(y\left|\omega ,\upsilon ,\beta \right.\right)p\left(\beta \right)p\left(\omega \left|\alpha \right.\right)p\left(\upsilon \left|\alpha \right.\right)p\left(\alpha \right)\\ & =\prod_{f=1}^{F}p(y^{f}|\omega^{f},\upsilon^{f},\beta^{f})\prod_{f=1}^{F}p(\beta^{f})\prod_{f=1}^{F}\prod_{n=1}^{N}p(\omega_{n}^{f}\left|\alpha_{n}\right.)\prod_{f=1}^{F}\prod_{n=1}^{N}p(\upsilon_{n}^{f}\left|\alpha_{n}\right.)\prod_{n=1}^{N}p(\alpha_{n}). \end{array}$$

**Figure 5:**
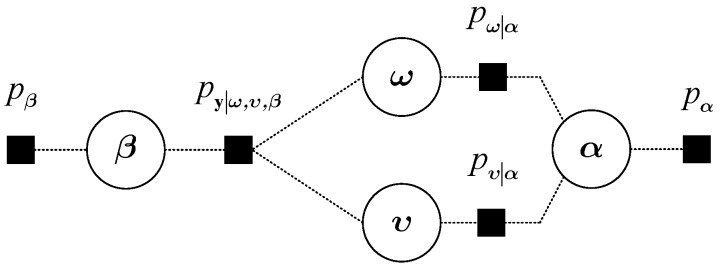
Factor graph representation of the joint PDF (22).

Figure 5 illustrates the factor graph representation of the joint PDF. In this figure, the circle nodes represent the random variables that the complicated global function relied on, while the square nodes represent the local functions.

For an arbitrary density function $q\left(z\right)$, the evidence $p\left(y\right)=\int p\left(y,z\right)dz$ can be decomposed as

(23) $$\ln p\left(y\right)=F\left(q;\Omega \right)+KL\left(q∥p\right),$$

where

(24) $$F\left(q;\Omega \right)=\int q\left(z\right)\ln\left(\frac{p\left(y,z;\Omega \right)}{q\left(z\right)}\right)dz,$$

(25) $$KL\left(q∥p\right)=-\int q\left(z\right)\ln\left(\frac{p\left(z\left|y;\Omega \right.\right)}{q\left(z\right)}\right)dz.$$

$F\left(q;\Omega \right)$ is a lower bound of $\ln p\left(y\right)$, and $KL\left(q∥p\right)$ represents the Kullback-Leibler divergence (KLD) between the approximated posterior $q\left(z\right)$ and the exact posterior $p\left(z\left|y;\Omega \right.\right)$. The proposed joint sparse recovery algorithm maximizes $\ln p\left(y\right)$ iteratively. At each iteration, we set $KL\left(q∥p\right)=0$ to minimize the KLD and update $q\left(z\right)$ accordingly. This will lead to the lower bound $F\left(q,\Omega \right)$ increasing to $\ln p\left(y\right)$. Meanwhile, the updating of q(z) may enlarge $\ln p\left(y\right)$ and lead to a new non-negative KLD. We will minimize the new KLD and update q(z) in the next iteration. By doing so, the log-likelihood $\ln p\left(y\right)$ will be maximized, and the approximated posterior $q\left(z\right)$ can be optimized iteratively.

However, $p\left(z\left|y;\Omega \right.\right)$ cannot be computed analytically. Thereby, directly updating $q\left(z\right)$ is intractable. To bypass this difficulty, we resort to the variational approximation method. It assumes that the posteriors of z are independent, i.e.,

(26) $$q\left(z\right)=q\left(\alpha \right)q\left(\omega \right)q\left(\upsilon \right)q\left(\beta \right)=q\left(\alpha \right)\prod_{f=1}^{F}q(\omega^{f})\prod_{f=1}^{F}q(\upsilon^{f})\prod_{f=1}^{F}q(\beta^{f}).$$

By applying the above assumption, in each iteration, the log-posterior of $z_{i}\in z$ can be approximated as the expectation of the joint PDF with respect to other hidden variables $\left(z-z_{i}\right)$. More specifically, the log-posteriors of z are approximated as

(27) $$\begin{array}{cc} \ln q(\omega^{f}) & ={\langle \ln p\left(y,z\right)\rangle }_{q\left(\alpha \right)\prod_{i\neq f}q\left(\omega^{i}\right)q\left(\upsilon \right)q\left(\beta \right)}+\xi , \end{array}$$

(28) $$\begin{array}{cc} \ln q(\upsilon^{f}) & ={\langle \ln p\left(y,z\right)\rangle }_{q\left(\alpha \right)q\left(\omega \right)\prod_{i\neq f}q\left(\upsilon^{i}\right)q\left(\beta \right)}+\xi , \end{array}$$

(29) $$\begin{array}{cc} \ln q(\beta^{f}) & ={\langle \ln p\left(y,z\right)\rangle }_{q\left(\alpha \right)q\left(\omega \right)q\left(\upsilon \right)\prod_{i\neq f}q\left(\beta^{i}\right)}+\xi , \end{array}$$

(30) $$\begin{array}{cc} \ln q(\alpha ) & ={\langle \ln p\left(y,z\right)\rangle }_{q\left(\omega \right)q\left(\upsilon \right)q\left(\beta \right)}+\xi , \end{array}$$

where ξ denotes a normalizing constant. It makes the corresponding $q\left(\cdot \right)$ a true PDF. The update rule for each hidden variable is derived below.

In (27), the terms independent of $\omega^{f}$ can be treated as a constant value. Keeping only the terms that are related to $\omega^{f}$, $\ln q(\omega^{f})$ can be given as

(31) $$\ln q\left(\omega^{f}\right)\propto {\langle \ln p\left(y^{f}|\omega^{f},\upsilon^{f},\beta^{f}\right)+\ln p\left(\omega^{f}|\alpha \right)\rangle }_{q\left(\alpha \right)q\left(\upsilon^{f}\right)q\left(\beta^{f}\right).}$$

Note that $q(\omega^{f})$ and $q(\upsilon^{f})$ follow the multivariate Gaussian distribution. We assume they have the following forms after the updating

(32) $$\begin{array}{cc} q(\omega^{f}) & =N\left(\omega^{f}|\mu_{\omega }^{f},\Sigma_{\omega }^{f}\right), \end{array}$$

(33) $$\begin{array}{cc} q(\upsilon^{f}) & =N\left(\upsilon^{f}|\mu_{\upsilon }^{f},\Sigma_{\upsilon }^{f}\right), \end{array}$$

where $\mu_{\omega }^{f}$ and $\mu_{\upsilon }^{f}$ are the mean vectors, $\Sigma_{\omega }^{f}$ and $\Sigma_{\upsilon }^{f}$ are the covariance matrices. The update of the posterior distribution is equivalent to seeking appropriate values for the parameters in the approximated posterior distribution. Our goal is to learn the values of the mean vectors and covariance matrices based on the prior distributions and likelihood function. Substituting (14) and (21) into (31), after some rearrangement, yields

(34) $$\begin{array}{c} \ln q\left(\omega^{f}\right)\propto -\frac{1}{2}{\left(\omega^{f}\right)}^{T}{\left(\Sigma_{\omega }^{f}\right)}^{-1}\omega^{f}+{\left(\omega^{f}\right)}^{T}{\left(\Sigma_{\omega }^{f}\right)}^{-1}\mu_{\omega }^{f}, \end{array}$$

where

(35) $$\begin{array}{cc} \Sigma_{\omega }^{f} & ={\left(\langle \beta^{f}\rangle \Psi^{T}\Psi +\langle \Lambda \rangle \right)}^{-1}, \end{array}$$

(36) $$\begin{array}{cc} \mu_{\omega }^{f} & =\langle \beta^{f}\rangle \Sigma_{\omega }^{f}\Psi^{T}\left(y^{f}-\Psi^{\prime }\mu_{\upsilon }^{f}\right). \end{array}$$

In the same manner, $\ln q(\upsilon^{f})$ can be given as

(37) $$\ln q\left(\upsilon^{f}\right)\propto {\langle \ln p\left(y^{f}|\omega^{f},\upsilon^{f},\beta^{f}\right)+\ln p\left(\upsilon^{f}\left|\alpha \right.\right)\rangle }_{q\left(\alpha \right)q\left(\omega^{f}\right)q\left(\beta^{f}\right).}$$

Substituting (15) and (21) into (37), after some rearrangement, yields

(38) $$\begin{array}{cc} \ln q(\upsilon^{f})\propto & -\frac{1}{2}{\left(\upsilon^{f}\right)}^{T}{\left(\Sigma_{\upsilon }^{f}\right)}^{-1}\upsilon^{f}+{\left(\upsilon^{f}\right)}^{T}{\left(\Sigma_{\upsilon }^{f}\right)}^{-1}\mu_{\upsilon }^{f}, \end{array}$$

where

(39) $$\begin{array}{cc} \Sigma_{\upsilon }^{f} & ={\left(\langle \beta^{f}\rangle {\Psi^{\prime }}^{T}\Psi^{\prime }+\langle \Lambda \rangle \right)}^{-1}, \end{array}$$

(40) $$\begin{array}{cc} \mu_{\upsilon }^{f} & =\langle \beta^{f}\rangle \Sigma_{\upsilon }^{f}{\Psi^{\prime }}^{T}\left(y^{f}-\Psi \mu_{\omega }^{f}\right). \end{array}$$

Keeping only the terms of (29) that are related to $\beta^{f}$, $\ln q(\beta^{f})$ can be given as

(41) $$\ln q\left(\beta^{f}\right)\propto {\langle \ln p\left(y^{f}|\omega^{f},\upsilon^{f},\beta^{f}\right)+\ln p\left(\beta^{f}\right)\rangle }_{q\left(\omega^{f}\right)q\left(\upsilon^{f}\right).}$$

We assume that the posterior of $\beta^{f}$ follows a Gamma distribution, i.e.,

(42) $$q\left(\beta^{f}\right)=\mathrm{Gamma}\left(\beta^{f}|{\tilde{a}}^{f},{\tilde{b}}^{f}\right),$$

where ${\tilde{a}}^{f}$ and ${\tilde{b}}^{f}$ denote the deterministic parameters of the updated posterior distribution. To infer them, we substitute (20) and (21) into (41). After some rearrangement, the posterior can be given as

(43) $$\ln q\left(\beta^{f}\right)\propto \left({\tilde{a}}^{f}-1\right)\ln\beta^{f}-{\tilde{b}}^{f}\beta^{f},$$

where

(44) a˜f=af+MM22,

(45) $$\begin{array}{cc} {\tilde{b}}^{f}= & \;b^{f}+\frac{1}{2}{∥y^{f}-\Psi \mu_{\omega }^{f}-\Psi^{\prime }\mu_{\upsilon }^{f}∥}_{2}^{2}+\frac{1}{2}\left(\mathrm{tr}\left({\Psi \Sigma }_{\omega }^{f}\Psi^{T}\right)+\mathrm{tr}\left({\Psi^{\prime }\Sigma }_{\upsilon }^{f}{\Psi^{\prime }}^{T}\right)\right). \end{array}$$

Similarly, we only keep the terms that are related to α in (30), and thereby $\ln q\left(\alpha \right)$ can be given as

(46) $$\ln q\left(\alpha \right)\propto {\langle \sum_{f=1}^{F}\ln p(\omega^{f}|\alpha )+\sum_{f=1}^{F}\ln p(\upsilon^{f}|\alpha )+\ln p\left(\alpha \right)\rangle }_{\prod_{f=1}^{F}q\left(\omega^{f}\right)\prod_{f=1}^{F}q(\upsilon^{f}).}$$

The approximated posterior of α is assumed to be a multivariate Gamma distribution, i.e.,

(47) $$q\left(\alpha \right)=\prod_{n=1}^{N}\mathrm{Gamma}(\alpha_{n}|\tilde{c},{\tilde{d}}_{n}),$$

where $\tilde{c}$ and ${\left\{{\tilde{d}}_{n}\right\}}_{n=1}^{N}$ are deterministic parameters. Substituting (14), (15) and (16) into (46), after some rearrangement, yields

(48) $$\ln q\left(\alpha \right)\propto \left(\tilde{c}-1\right)\sum_{n=1}^{N}\ln\alpha_{n}-\sum_{n=1}^{N}{\tilde{d}}_{n}\alpha_{n},$$

where

(49) $$\begin{array}{cc} \tilde{c} & =c+F, \end{array}$$

(50) $$\begin{array}{cc} {\tilde{d}}_{n} & =d_{n}+\frac{1}{2}\sum_{f=1}^{F}\left({\left[\mu_{\omega }^{f}\right]}_{n}^{2}+{\left[\Sigma_{\omega }^{f}\right]}_{n,n}+{\left[\mu_{\upsilon }^{f}\right]}_{n}^{2}+{\left[\Sigma_{\upsilon }^{f}\right]}_{n,n}\right). \end{array}$$

The notation ${\left[\cdot \right]}_{n}$ denotes the *n*-th component of the input vector, and ${\left[\cdot \right]}_{n,n}$ denotes the (n,n)-th entry of the input matrix. Based on the results of posterior inference, the required expectations can be calculated as

(51) 〈βf〉=a˜fa˜fb˜fb˜f,f=1,2,...,F.

(52) $$\begin{array}{c} \langle \Lambda \rangle =\mathrm{diag}\left(\langle \alpha_{1}\rangle ,\langle \alpha_{2}\rangle ,...,\langle \alpha_{N}\rangle \right), \end{array}$$

where

(53) αn=c˜c˜d˜nd˜n,n=1,2,...,N.

### 4.3. Joint Sparse Reconstruction

According to the above update rules, we can successively update the posteriors of hidden variables z. The jointly sparse vectors S can be reconstructed according to these posteriors. The algorithm of reconstructing S is summarized as follows:

1. For $f\in \left\{1,2,...,F\right\}$, update $q(\omega^{f})$ by using (35) and (36); update $q(\upsilon^{f})$ by using (39) and (40). $\langle \beta^{f}\rangle$ and $\langle \Lambda \rangle$ are obtained based on the current posteriors of $\beta^{f}$ and α.
2. For $f\in \left\{1,2,...,F\right\}$, update $q(\beta^{f})$ according to (44), (45) and the current posteriors of $\omega^{f}$ and $\upsilon^{f}$.
3. Update $q\left(\alpha \right)$ according to (49), (50) and the current posteriors of ω and υ.
4. If a convergence criterion has been met, terminate and choose the posterior means of ω and υ as the estimation of S. Otherwise, go to step 1).

Based on the above joint sparse recovery algorithm, we can obtain a reconstructed sparse vector set $\hat{S}={\left\{\mu_{\omega }^{f},\mu_{\upsilon }^{f}\right\}}_{f=1}^{F}$. The computational cost of the algorithm is dominated by the matrix inversion operations in (35) and (39), whose computational complexities are $O\left(N^{3}F\right)$. Moreover, the matrix-vector multiplications in (36) and (40), as well as the matrix multiplication operations in (45) can also incur heavy computational burden. Their computational complexities are $O\left(N^{2}MF\right)$. Thus, when applying ComDec in large-scale areas (where *N* is large), the joint sparse recovery algorithm will be computationally expensive. In this paper, we adopt the following four methods to alleviate the computational burden of the proposed algorithm.

In step 1, the covariance matrices $\Sigma_{\omega }^{f}$ and $\Sigma_{\upsilon }^{f}$ are computed by (35) and (39), which contain matrix inversion operations. Using the matrix inversion lemma [30], the covariance matrices can be evaluated as

(54) $$\begin{array}{cc} \Sigma_{\omega }^{f} & ={\langle \Lambda \rangle }^{-1}-{\langle \Lambda \rangle }^{-1}\Psi^{T}{\left(\Xi_{\omega }^{f}\right)}^{-1}\Psi {\langle \Lambda \rangle }^{-1}, \end{array}$$

(55) $$\begin{array}{cc} \Sigma_{\upsilon }^{f} & ={\langle \Lambda \rangle }^{-1}-{\langle \Lambda \rangle }^{-1}{\Psi^{\prime }}^{T}{\left(\Xi_{\upsilon }^{f}\right)}^{-1}\Psi^{\prime }{\langle \Lambda \rangle }^{-1}, \end{array}$$

where

(56) $$\begin{array}{cc} \Xi_{\omega }^{f} & =\Psi {\langle \Lambda \rangle }^{-1}\Psi^{T}+{\langle \beta^{f}\rangle }^{-1}I_{M}, \end{array}$$

(57) $$\begin{array}{cc} \Xi_{\upsilon }^{f} & =\Psi^{\prime }{\langle \Lambda \rangle }^{-1}{\Psi^{\prime }}^{T}+{\langle \beta^{f}\rangle }^{-1}I_{M}. \end{array}$$

With these matrix transformations, we only need to compute the inversions of $\Xi_{\omega }^{f}$ and $\Xi_{\upsilon }^{f}$, whose computational complexities are $O\left(M^{3}\right)$(M≪N). Note that, $\langle \Lambda \rangle$ is an N×N diagonal matrix. We can easily obtain its inversion.

As mentioned before, the expectation evaluations of $\mu_{\omega }^{f}$ and $\mu_{\upsilon }^{f}$ can also lead to a high computational cost. To reduce their computational complexities, we reformulate (36) and (40) to cast them as a problem of solving the following linear systems of equations:

(58) $$\begin{array}{cc} \left(\Psi^{T}\Psi +\frac{\langle \Lambda \rangle }{\langle \beta^{f}\rangle }\right)\mu_{\omega }^{f} & =\Psi^{T}\left(y^{f}-\Psi^{\prime }\mu_{\upsilon }^{f}\right), \end{array}$$

(59) $$\begin{array}{cc} \left({\Psi^{\prime }}^{T}\Psi^{\prime }+\frac{\langle \Lambda \rangle }{\langle \beta^{f}\rangle }\right)\mu_{\upsilon }^{f} & ={\Psi^{\prime }}^{T}\left(y^{f}-\Psi \mu_{\omega }^{f}\right). \end{array}$$

The equations can be solved by the conjugate gradient (CG) algorithm. Theoretically, we can reach the exact solution after *N* iterations. However, this may incur a considerable computational burden. Fortunately, in practice, a few iterations is sufficient to obtain a good solution. If *W*$\left(W\ll N\right)$ iterations are required, the computational complexity of the CG algorithm will be $O\left(WN\log N\right)$.

In step 2, the computational cost is mainly attributed to the matrix multiplications in the last term of (45). To mitigate this, (45) can be rewritten as

(60) $${\tilde{b}}^{f}=\;b^{f}+\frac{1}{2}{∥y^{f}-\Psi \mu_{\omega }^{f}-\Psi^{\prime }\mu_{\upsilon }^{f}∥}_{2}^{2}+\frac{1}{\langle \beta^{f}\rangle }\sum_{n=1}^{N}\left(1-\frac{\langle \alpha_{n}\rangle }{2}\left({\left[\Sigma_{\omega }^{f}\right]}_{n,n}+{\left[\Sigma_{\upsilon }^{f}\right]}_{n,n}\right)\right).$$

Here, matrix multiplications are replaced by simple element-wise operations. As a consequence, the computational complexity of step 2 can be reduced to $O\left(NMF\right)$. By using the CG method and the above reformulations, the computational cost per iteration of the proposed joint sparse recovery algorithm can be reduced to $O\left(NM^{2}F\right)$.

To further reduce the computational load and speed up the convergence, we conduct real-time grid pruning according to the posterior of α. In each iteration, when $\alpha_{n}$ is sufficiently large, ${\left\{\omega_{n}^{f},\upsilon_{n}^{f}\right\}}_{f=1}^{F}$ will be driven to zero. This imply that the contribution of grid *n* to the signal power fading is negligible. In this case, we can remove grid *n* from the grid set Π, which is defined as the set of grids where a target possibly exists. With the reduction of grids in Π, the computational load of the next iteration will decrease.

We denote the initial grid set by $\Pi^{\left(0\right)}≜\left\{1,2,...,N\right\}$. Then, the grid set in each iteration can be updated as

(61) $$\Pi^{\left(\tau \right)}=\Pi^{\left(\tau -1\right)}-\left\{n\left|\langle \alpha_{n}\rangle \right.>\alpha_{\mathrm{th}}\right\},$$

where τ denotes the iteration number and $\alpha_{\mathrm{th}}$ is the threshold of $\alpha_{n}$. For the reconstruction of S, the selection of $\alpha_{\mathrm{th}}$ provides a trade-off between the localization accuracy and the convergence speed. After grid pruning, the dimension of θ can be reduced to $N=|\Pi^{\left(\tau \right)}|$. In addition, α, S, Ψ, and $\Psi^{\prime }$ also should be pruned accordingly.

By virtue of the local convergence property of variational Bayesian inference [20], the proposed joint sparse recovery algorithm is guaranteed to be convergent. The stop criterion is set as the residual error Res smaller than a pre-determined threshold $r_{\mathrm{th}}$, where the Res is defined as

(62) $$Res=\sum_{f=1}^{F}{∥y^{f}-\Psi \mu_{\omega }^{f}-\Psi^{\prime }\mu_{\upsilon }^{f}∥}_{2}^{2}.$$

Furthermore, to prevent the computational load from being excessively high, we set a maximum iteration number $\tau_{\max}$. In this situation, the iterative algorithm will stop when Res becomes smaller than $r_{\mathrm{th}}$ or τ reaches $\tau_{\max}$.

### 4.4. Target Counting and Localization

Based on $\hat{S}$, the target number and locations can be estimated. However, these vectors are not strictly sparse. They may contain many negligible but nonzero coefficients. Thus, we estimate their common support by the following formulae:

(63) $$\hat{T}=\left\{n\left|20\mathrm{lg}\left(\frac{{\left[\mu_{\omega }^{\hat{f}}\right]}_{n}}{\max_{i\in \Pi }{\left[\mu_{\omega }^{\hat{f}}\right]}_{i}}\right)⩾\mu_{\mathrm{th}},\;n\in \Pi \right.\right\},$$

where

(64) $$\hat{f}=\arg\min_{f}{∥y^{f}-\Psi \mu_{\omega }^{f}-\Psi^{\prime }\mu_{\upsilon }^{f}∥}_{2}^{2}.$$

The common support of the jointly sparse vectors is estimated based on $\mu_{\omega }^{\hat{f}}$, in which $\hat{f}$ is the channel that has the minimum residual error. In (63), $\mu_{\mathrm{th}}$ is the sparsity threshold. We use it to filter out the small coefficients in $\mu_{\omega }^{\hat{f}}$. After that, we can estimate the target number and locations as $\hat{K}=\left|\hat{T}\right|$ and $\hat{L}=\left\{\left(x_{n},y_{n}\right)|n\in \hat{T}\right\}$, respectively.

## 5. Numerical Evaluation

### 5.1. Simulation Setup

The numerical simulations are carried out in MATLAB R2015b 64 bit version running on a PC with i7-8550U and 16 GB memory. In our simulations, the channel state information is assumed to be collected from the wireless devices that implement an orthogonal frequency division multiplexing (OFDM) system. The wireless devices are assumed to mode at 2.4 GHz with 20 MHz bandwidth. Table 1 summarizes the default values of simulation parameters. The numerical simulations are conducted in a complex radio transmission environment. In this transmission environment, as the impact of environmental dynamics, the environmental parameters are changing with time. Moreover, the measurement of each channel is corrupted by addictive white Gaussian noise. The signal-to-noise ratio (SNR) of each channel is defined as

(65) $$\mathrm{SNR}=10\mathrm{lg}\frac{∥\Phi^{f}\theta {∥}_{2}^{2}}{M{\left(\sigma^{f}\right)}^{2}},\;\;f=1,2,...,F,$$

where $\sigma^{f}$ is the standard deviation of the measurement noise vector.

In our simulations, all results are averaged over $T=10^{3}$ Monte Carlo trials. For each trial, the localization error $E_{t}$ is computed as

(66) $$E_{t}=\frac{\sum_{k=1}^{\min\{K,\hat{K}\}}{∥L_{k}-{\hat{L}}_{k}∥}_{2}}{\min\{K,\hat{K}\}},$$

where $L_{k}$ and ${\hat{L}}_{k}$ represent the true and estimated Cartesian coordinates of target *k*. We use the “average localization error” (Avg.Error) and “root-mean-square error” (Rms.Error) as the metrics to measure the localization accuracy. The Avg.Error is defined as

(67) $$Avg.Error=\frac{\sum_{t=1}^{T}E_{t}}{T}.$$

The Rms.Error is defined as

(68) $$Rms.Error=\sqrt{\frac{\sum_{t=1}^{T}E_{t}^{2}}{T}}.$$

**Figure 6:**
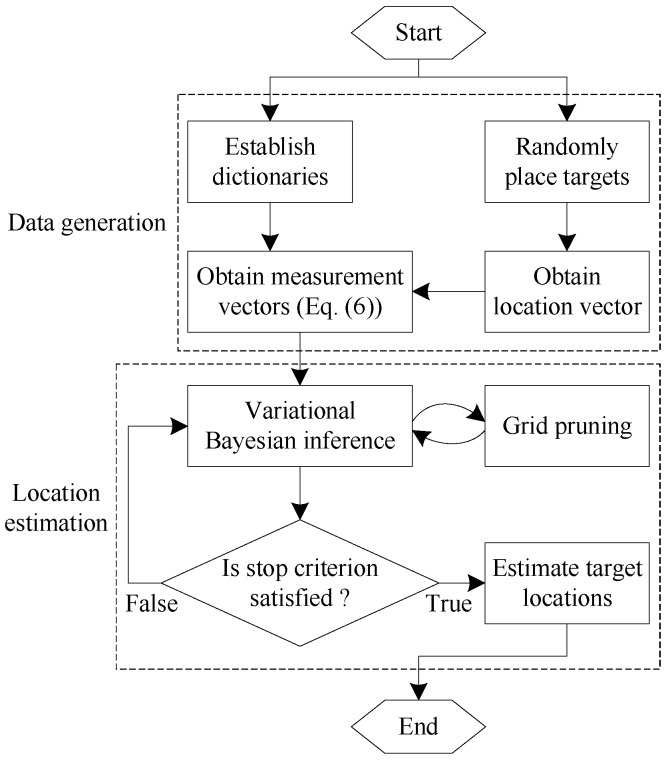
Simulation flowchart.

To evaluate the target counting performance, we introduce another metric Prob.CoC. It is the probability of correct counting (i.e., $\hat{K}=K$). We compare the performances of ComDec with other three CS-based DFL approaches: (i) LCS with the GMP algorithm (LCS-GMP) as the CS recovery algorithm [22], (ii) E-HIPA with the adaptive OMP algorithm (E-HIPA-OMP) as the CS recovery algorithm [23], and (iii) DR-DFL with the VEM algorithm for dictionary refinement and sparse recovery (DR-DFL-VEM) [14]. Table 2 reports the computational complexities and localization accuracies of multiple DFL methods. As can be seen from this table, the ComDec method can achieve the lowest average localization error (Avg.Error). It can enhance the accuracy and robustness of CS-based DFL by exploiting channel diversity. The computational complexity of the ComDec method is higher than the LCS and E-HIPA methods. The inferiority is mainly attributed to the estimation of the posterior distributions of sparse vectors. It noteworthy that the E-HIPA and LCS methods reconstruct the unknown sparse vector by using the OMP and GMP algorithms, respectively. They are greedy algorithms for sparse recovery, and can only provide a point estimation of the unknown sparse signal. In contrast, the proposed sparse recovery algorithm and VEM algorithm reconstruct sparse signal from a Bayesian perspective. They can provide a posterior belief (distribution function) for the values of the sparse signal, and therefore can achieve an improved accuracy. In real deployment, the sparse recovery algorithm is usually conducted on a server, where the computational resources are abundant. The server collects CSI measurements from all wireless nodes and estimates the target location accordingly. The simulation flowchart is shown in Figure 6. In the upper part of the flowchart, the measurement data is generated. In the lower part of the flowchart, the target locations can be estimated.

### 5.2. Impact of the Number of Channels

In this subsection, the impact of channel number on the performance of ComDec is evaluated. Theoretically, the accuracy of joint sparse recovery is closely related to the channel number. If we increase *F*, more useful information will be provided. Consequently, the target counting and localization performance will be improved.

**Figure 7:**
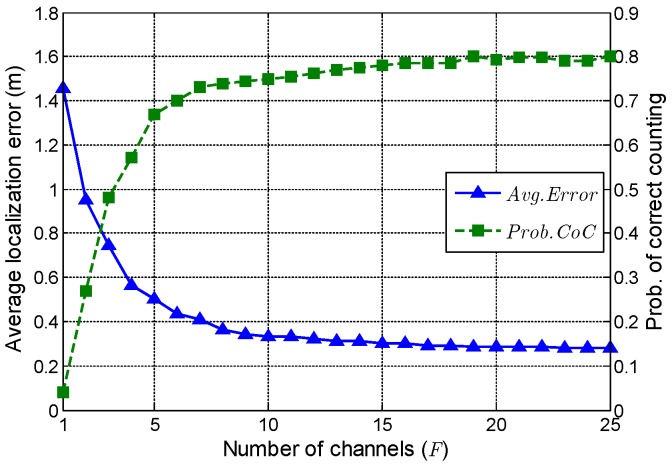
The performance of ComDec when channel number varies from 1 to 25.

In Figure 7, *F* varies from 1 to 25, and the Avg.Error of ComDec decreases as *F* increases. It is noteworthy that, the Avg.Error decreases dramatically when F<10, while decreases slowly when F>10. As in the case of Avg.Error, the Prob.CoC also increases as *F* increases, and its growth rate drops gradually. This is because the increase of *F* leads to a more complex hierarchical prior model, which contains (3F+1) hidden variables. When reconstructing S, the posterior distributions of these hidden variables should be inferred. Thus, a large *F* may deteriorate the accuracy of the posterior inference. From this perspective, increasing the channel number has a negative effect on sparse recovery. In fact, when F>20, the negative effect of increasing *F* will offset the positive effect contributed by channel diversity. Hence, we set F=15 in our simulation to achieve a trade-off between model simplicity and the performance of target counting and localization.

### 5.3. Effectiveness of ComDec in Changing Environments

To mitigate the dictionary mismatches introduced by environmental dynamics, the proposed ComDec method combines the environmental parameters and the location vector to form a set of jointly sparse vectors. We treat these vectors as random variables and learn their values by variational Bayesian inference. This makes the dictionary parameters can adapt to the environmental changes and different channel characteristics. In this subsection, we test the effectiveness and robustness of ComDec in changing environments.

**Figure 8:**
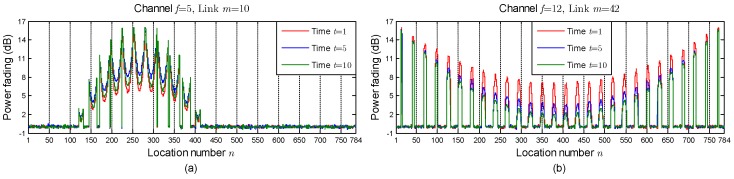
The values of the dictionary atoms: (**a**) $\phi ._{10}^{5}$; (**b**) $\phi_{42}^{12}$, when t=1, 5, and 10.

Firstly, we set up a simulated changing environment. To simulate the environmental dynamics in changing environments, we add Gaussian noises to the environmental parameters ($\gamma^{f}$ and $\rho^{f}$). Based on these noisy environmental parameters and the saddle surface model, the dictionaries ${\left\{\Phi^{f}\right\}}_{f=1}^{F}$ can be built. After that, we can obtain the measurement vectors ${\left\{y^{f}\right\}}_{f=1}^{F}$ according to (6). In our simulation, the values of $\gamma^{f}$ and $\rho^{f}$ are calculated by

(69) $$\begin{array}{cc} \gamma_{t}^{f} & =\gamma_{0}^{f}+\sum_{i=1}^{t}\varpi_{\gamma }^{i},\;\;f=1,2,...,F, \end{array}$$

(70) $$\begin{array}{cc} \rho_{t}^{f} & =\rho_{0}^{f}+\sum_{i=1}^{t}\varpi_{\rho }^{i},\;\;f=1,2,...,F, \end{array}$$

where $\varpi_{\gamma }^{i}$ and $\varpi_{\rho }^{i}$ are additive white Gaussian noises whose variances are 0.6 and 0.06, respectively. The setting of the noise variances is according to the real data in changing environments. *t* represents the times of environmental changes and also denotes the number of additive noises added to the original environmental parameters ($\gamma_{0}^{f}$ and $\rho_{0}^{f}$). In our simulations, we set $\gamma_{0}^{f}=17.0831$ and $\rho_{0}^{f}=0.35122$. In addition, uncorrelated Gaussian noises are added to the dictionaries ${\left\{\Phi^{f}\right\}}_{f=1}^{F}$ to produce an SNR of 40 dB. Figure 8 depicts the variations of two dictionary atoms in the simulated changing environment. As expected, the accumulation of additive noises can lead to a deviation of dictionary atoms, which is a simulation of the effect of environmental dynamics in real-world environments.

**Figure 9:**
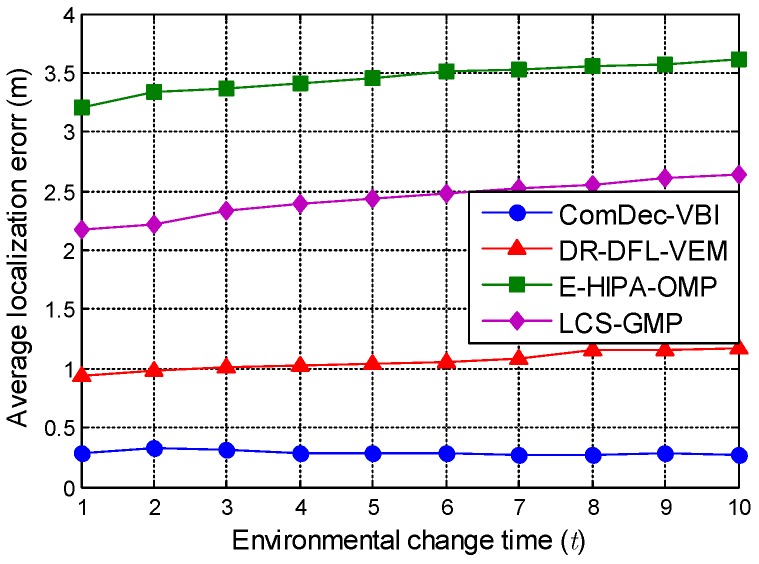
Effect of environmental changes on the average localization error (Avg.Error) of multiple DFL approaches.

Secondly, in the simulated changing environment, the localization performances of multiple CS-based multi-target DFL methods are evaluated. Figure 9 compares the localization performances when *t* increases from 1 to 10. The proposed ComDec method that using the variational Bayesian inference technique for joint sparse recovery (ComDec-VBI) can achieve the minimum Avg.Error. It should be noted that, its localization performance is robust to the environmental changes. In contrast, with the increase of *t*, the localization accuracies of other three DFL methods deteriorate gradually. This reveals that these methods are sensitive to environmental dynamics. The simulation results demonstrate the impact of environmental dynamics on Avg.Error and verify the accuracy and robustness of the ComDec method.

**Figure 10:**
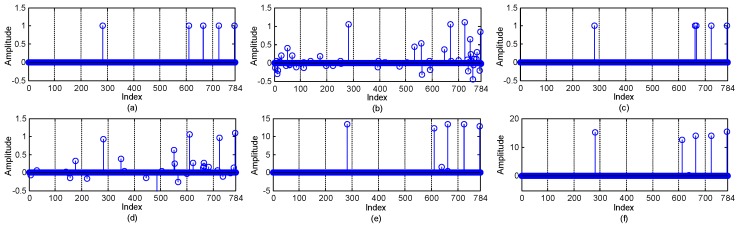
Comparison of the original location vector and the reconstructed sparse vectors of different DFL approaches. (**a**) the original location vector; The reconstructed sparse vector corresponding to (**b**) E-HIPA; (**c**) LCS; (**d**) DR-DFL; (**e**) ComDec (F=5); (**f**) ComDec (F=15).

Finally, we investigate the sparse reconstruction performances of the CS recovery algorithms that adopted in the CS-based DFL methods. We run E-HIPA, LCS, DR-DFL, and the ComDec method with F=5 and F=15 to reconstruct the target location vector. Figure 10 shows an example of the recovered sparse vectors of different DFL approaches. As can be seen, the recovered sparse vectors corresponding to E-HIPA and DR-DFL have many negligible but nonzero coefficients. Although the reconstructed sparse vector in Figure 10c does not have small coefficients, the indices of the nonzero components are different from those of the original location vector. Fortunately, the recovered sparse vector corresponding to our proposed ComDec method has a few small coefficients, and the indices of their significant coefficients are the same as those in the original location vector. Based on them, the target number and target locations can be estimated correctly.

**Figure 11:**
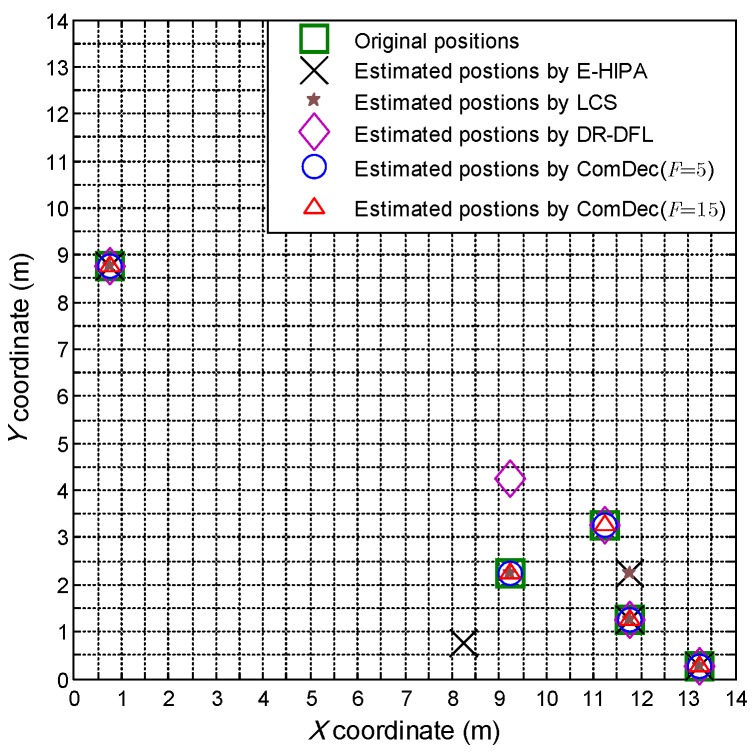
Comparison of the original target positions and the estimated target positions of different DFL approaches.

Figure 11 shows the target positions estimated by multiple CS-based DFL methods. As can be seen, the ComDec method can accurately estimate target positions, whereas other DFL methods have 1 or 2 incorrect estimated positions. This is because the proposed ComDec method can eliminate the position ambiguities introduced by environmental dynamics by taking advantage of the channel diversity.

### 5.4. Localization Error vs. SNR

**Figure 12:**
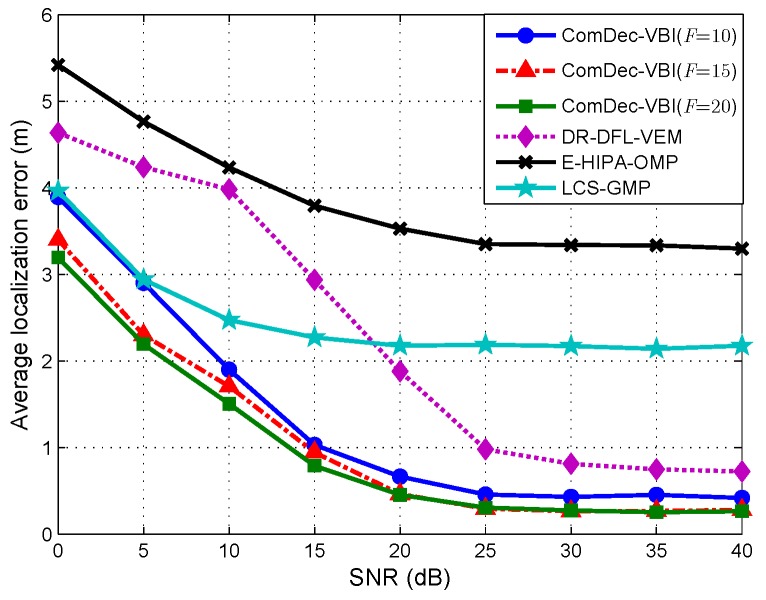
Average localization error with different SNR.

In this subsection, the localization performances of different DFL methods under different SNRs are evaluated. In cellular communication (e.g., LTE), the typical SNR value is below 0 dB. However, in short range communication protocols such as 802.11 b/g, the typical SNR value is from 10–15 dB (low signal) to 40 dB (excellent signal). In fact, the scenario that is chosen for our simulations is the short-range communication scenario. Thus, the SNR in this simulation is varied from 0 dB to 40 dB. Figure 12 shows the Avg.Error of the ComDec method (F=10, 15, and 20) and other DFL methods. As can be seen, the Avg.Error of all DFL methods decrease with the increase of SNR. Generally, the localization error is mainly caused by the measurement noise and the environmental dynamics in changing environments. As the ComDec and DR-DFL have countermeasures to mitigate the dictionary mismatches introduced by environmental dynamics, their Avg.Error decrease dramatically when SNR increases from 0 dB to 25 dB.

Furthermore, as can be seen from Figure 12, by increasing the channel number, the accuracy improvement of ComDec in low SNR case (SNR < 20 dB) is much larger than the improvement in high SNR case (SNR > 20 dB). This demonstrates that the channel diversity can help to reduce the location uncertainty that introduced by measurement noises especially in the low SNR case. Moreover, the DR-DFL performs better than LCS in the high SNR case, while performs worse in low SNR cases. This is because, in the low SNR case, the measurement noise can significantly affect the dictionary refinement process. It may lead to a wrong estimation of the dictionary parameters, and the dictionary mismatches will deteriorate the localization performance seriously. Fortunately, in the high SNR case, the DR-DFL can correctly estimate the dictionary parameters and performs better than LCS.

### 5.5. Localization Error vs. Number of Targets

**Figure 13:**
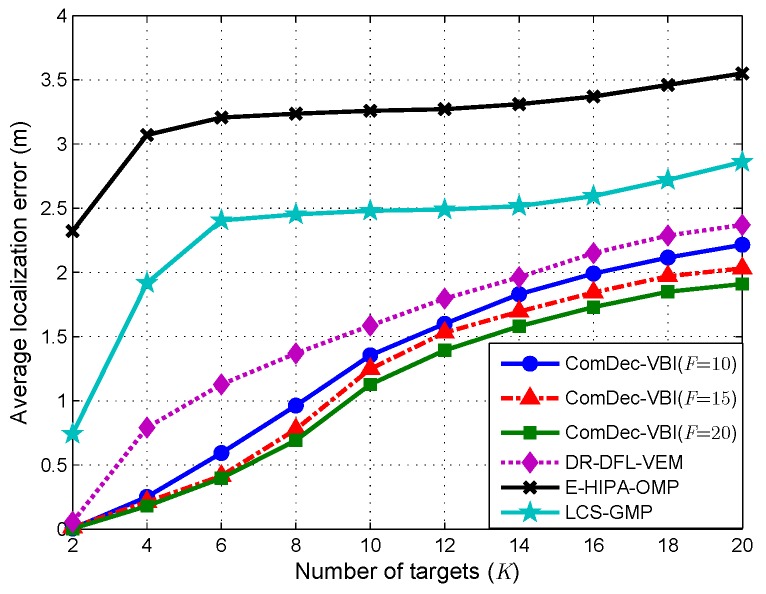
Average localization error with different *K*.

In this subsection, we examine the performances of multiple CS-based DFL methods when *K* varies from 2 to 20 at a step of 2. In this simulation, we set the environmental change time t=1 to simulate the environmental dynamics in changing environments. With the increase of *K*, the joint sparsity level of the jointly sparse vectors S changes accordingly. This will make the localization accuracies of all CS-based DFL decrease. Figure 13 plots the Avg.Error as a function of *K*. As we can see, the Avg.Error of all DFL methods increase as *K* increases, and the ComDec is more accurate than other DFL methods. Furthermore, we can also observe that the DR-DFL and ComDec perform better than the E-HIPA and LCS. This verifies that the Bayesian recovery algorithm can provide a more accurate sparse reconstruction than the greedy algorithm for CS recovery.

## 6. Conclusions and Future Work

In this paper, a novel multi-channel CS-based DFL method, ComDec is proposed. It can solve the dictionary mismatch problem caused by environmental dynamics in changing environments. The key novelty of ComDec is the making use of the channel diversity of CSI measurements in CS-based DFL. Moreover, the dictionary parameters of multiple channels are assumed to be adjustable. They can adapt to the changes of environment and different channel characteristics. In this manner, we can avoid the site survey process that is typically adopted in fingerprinting-based DFL and improve the robustness of DFL against environmental dynamics. We formulate the CS-based DFL problem as a joint sparse recovery problem, and develop a novel joint sparse recovery algorithm under the variational Batesian inference framework. Furthermore, four methods are presented to reduce its computational complexity. Finally, numerical simulation results validate the effectiveness of the ComDec method.

As future work, we will further investigate the dictionary mismatch problem in CS-based multi-target DFL caused by more realistic and complex environmental dynamics. Additionally, we intend to design and implement an efficient and accurate DFL framework that can utilize the phase information of CSI measurements.

## Figures and Tables

**Table 1:** Default values of simulation parameters.

Parameters	Explains	Values
*F*	number of channels	15
*K*	number of targets	5
*M*	number of links	56
*N*	number of grids	784
*l*	link length	14 m
*W*	iteration number of CG algorithm	17
$\alpha_{\mathrm{th}}$	pruning threshold	10
$\tau_{\max}$	maximum number of iteration	600
$r_{\mathrm{th}}$	residual error threshold	$10^{-4}$
$\mu_{\mathrm{th}}$	sparsity threshold	−20 dB

**Table 2:** Computational complexity and localization accuracy.

DFL Method	Sparse Recovery Algorithm	Computational Complexity	Avg.Error	Rms.Error
ComDec	VBI	$O(NM^{2}F)$	0.296 m	0.612 m
DR-DFL	VEM	$O(MN^{2})$	0.981 m	1.420 m
E-HIPA	OMP	O(KMN+log(2N))	3.351 m	3.509 m
LCS	GMP	O(MN)	2.188 m	2.462 m

## Abbreviations

The following abbreviations are used in this manuscript:

DFL	Device-free localization
VBI	Variational Bayesian inference
LBS	Location-based service
OFDM	Orthogonal Frequency Division Multiplexing
LTE	Long Term Evolution
SNR	Signal-to-noise ratio
CS	Compressive sensing
RIP	Restricted isometry property
RTI	Radio tomographic imaging
GMP	Greedy matching pursuit
LCS	Device-free localization based on compressive sensing
PDF	Probability density function
CG	Conjugate gradient
OMP	Orthogonal matching pursuit
E-HIPA	The energy-efficient framework for high-precision multi-target-adaptive device-free localization
VEM	Variational expectation-maximization
DR-DFL	Dictionary refinement based device-free localization
ComDec	Compressive sensing-based multi-target device-free localization
M-OMP	Multiple measurement vector orthogonal matching pursuit
MMV	Multiple measurement vector
COTS	Commercial off-the-shelf
RSS	Received signal strength
WSN	Wireless sensor networks
CSI	Channel state information
LASSO	Least absolute shrinkage and selection operator
LOS	Line-of-sight
M-SBL	Multiple sparse Bayesian learning
KLD	Kullback-Leibler divergence
M-BMP	Multiple measurement vector basic matching pursuit
